# Design, Synthesis, and Cytotoxicity Evaluation of Novel Griseofulvin Analogues with Improved Water Solubility

**DOI:** 10.1155/2017/7386125

**Published:** 2017-12-07

**Authors:** Ahmed K. Hamdy, Mahmoud M. Sheha, Atef A. Abdel-Hafez, Samia A. Shouman

**Affiliations:** ^1^Department of Medicinal Chemistry, Faculty of Pharmacy, Assiut University, Assiut 71526, Egypt; ^2^Cancer Biology Department, National Cancer Institute, Cairo University, Cairo, Egypt

## Abstract

Griseofulvin** 1** is an important antifungal agent that has recently received attention due to its antiproliferative activity in mammalian cancer cells. Study of SAR of some griseofulvin analogues has led to the identification of 2′-benzyloxy griseofulvin** 3**, a more potent analogue which retards tumor growth through inhibition of centrosomal clustering. However, similar to griseofulvin** 1**, compound** 3** exhibited poor aqueous solubility. In order to improve the poor water solubility, six new griseofulvin analogues** 5**–**10** were synthesized and tested for their antiproliferative activity and water solubility. The semicarbazone** 9** and aminoguanidine** 10** analogues were the most potent against HCT116 and MCF-7 cell lines. In combination studies, compound** 9** was found to exert synergistic effects with tamoxifen and 5-fluorouracil against MCF-7 and HCT116 cells proliferation, respectively. The flow cytometric analysis of effect of** 9** on cell cycle progression revealed G2/M arrest in HCT116. In addition, compound** 9** induced apoptosis in MCF-7 cells. Finally, all synthesized analogues revealed higher water solubility than griseofulvin** 1** and benzyloxy analogue** 3** in pH 1.2 and 6.8 buffer solutions.

## 1. Introduction

Griseofulvin** 1**, a natural product from* Penicillium griseofulvum,* was first discovered in 1939 and has been known for its antifungal properties in guinea pigs and man since 1958 [1–4]. In 1968, griseofulvin** 1** was found to have an inhibitory effect on skin tumor induced by croton oil in mice [5] and to inhibit, alone or associated with other anticancer drugs, the* in vitro* proliferation of cancer cell lines [6, 7]. In addition, griseofulvin** 1** exhibits a lack of significant toxicity in humans and appears to selectively target tumor cells and spare healthy tissues [6, 8, 9]. The mode of action of griseofulvin** 1** has been the subject of large research efforts, where it was reported that** 1** binds to tubulin [10], inhibits tubulin polymerization, and disturbs microtubule dynamics [11, 12]. The selectivity of** 1** against tumor cells is due to its ability to inhibit centrosomal clustering* in vitro *[13]. While normal cells have exactly two centrosomes at the onset of mitosis, cancer cells often have multiple centrosomes that lead to formation of multiple spindle poles. To avoid lethal multipolar mitosis during cell divisions, cancer cells rely on a dynamic process called centrosomal clustering to form pseudobipolar spindles and thus ensure appropriate cell division. Consequently, inhibition of centrosomal clustering may constitute a novel therapeutic target for selective eradication of cancer cells with multiple centrosomes [13–15].

Several griseofulvin analogues with structural modification at 4, 5, 6, 2′, 3′, and 4′ positions were synthesized and tested for activity against some cancer cell lines. The benzyloxy analogue** 3** was found to be the most potent against MDA-MB-231 and SCC 114 cell lines [16, 17] with a 25-fold increase in activity as a centrosome clustering inhibitor compared to** 1**. In addition, it was reported that the benzyloxy analogue** 3** retards tumor growth in murine xenograft models of colon cancer and multiple myeloma through* in vivo* inhibition of centrosomal clustering [17, 18].

On the other hand, previous reports have revealed** 1** to be irregularly and incompletely absorbed from the gastrointestinal tract of man and laboratory animals. The incomplete absorption appears to be a result of the slow rate of dissolution of griseofulvin in the gastrointestinal fluids due to its extremely low solubility in water [19]. We herein report the synthesis and biological evaluation of six new griseofulvin analogues** 5**–**10** with different polar moieties at position 4′. In addition to biological activity, griseofulvin** 1** and analogues hereof were also subjected to solubility study at simulated gastric (pH 1.2) and intestinal (pH 6.8) buffer solutions.

## 2. Results and Discussion

### 2.1. Chemistry

**Scheme 1:**
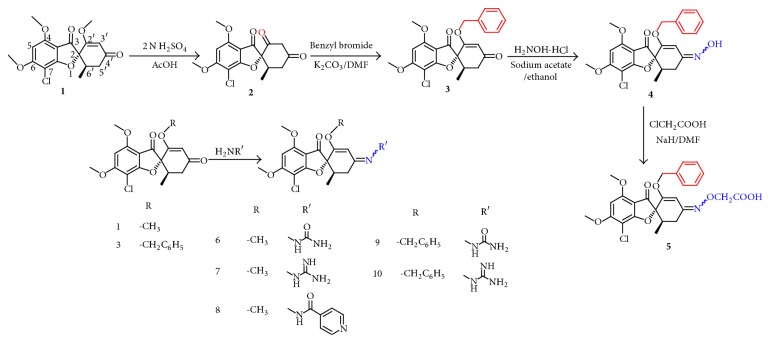
Synthesis of the target compounds** (5**–**10)**.

The target compounds** 5**–**10** and intermediates** 2**–**4** were prepared as outlined in Scheme 1. Griseofulvin acid** 2** was synthesized as reported [20] through hydrolysis of griseofulvin** 1**. Alkylation of** 2** with benzyl bromide in presence of anhydrous potassium carbonate gave 2′-benzyloxy analogue** 3**. For the preparation of the oxime derivative** 4**, a mixture of** 3** and hydroxylamine hydrochloride was refluxed in presence of anhydrous sodium acetate [17]. The carboxymethoxime analogue** 5** was synthesized through alkylation of** 4** with chloroacetic acid. The Schiff bases** 6**–**10** were obtained through reflux of either** 1** or** 3** with appropriate amine.

The prepared compounds were identified by IR, ^1^H-NMR, ^13^C-NMR, and elemental analysis. All compounds gave satisfactory analytical and spectroscopic data, which were in full accordance with their depicted structures.

### 2.2. Biological Investigations

#### 2.2.1. Antiproliferative Activity

The growth inhibitory effect caused by griseofulvin analogues** 5**–**10** on human breast cancer cell line MCF-7 and human colon cancer cell line HCT116 in comparison to** 1**, the benzyloxy analogue** 3**, tamoxifen, and 5-fluorouracil was evaluated using the Sulforhodamine B (SRB) assay after 72-hour exposure. From the results in Table 1, it is obvious that all tested analogues exhibited improved antiproliferative activity compared to** 1** against both cancer cell lines. Analogues** 9** and** 10** were the most potent with 2-fold increase in activity over 5-fluorouracil against HCT116 and comparable activity to tamoxifen against MCF-7 cells. The carboxymethoxime analogue** 5** revealed a higher cytotoxic activity than** 1** and** 3** against MCF-7 and weak activity against HCT116. From the results, it can be deduced that griseofulvin analogues** 5**–**10** suppress cell proliferation in a dose-dependent manner in MCF-7 and HCT116 cells. Formation of Schiff bases at 4′-carbonyl group of** 1** and** 3** with different polar hydrophilic moieties, especially semicarbazide and aminoguanidine, increased the anticancer activity.

#### 2.2.2. Compound** 9** Synergizes Antitumor Activity of Tamoxifen and 5-Fluorouracil

**Figure 1:**
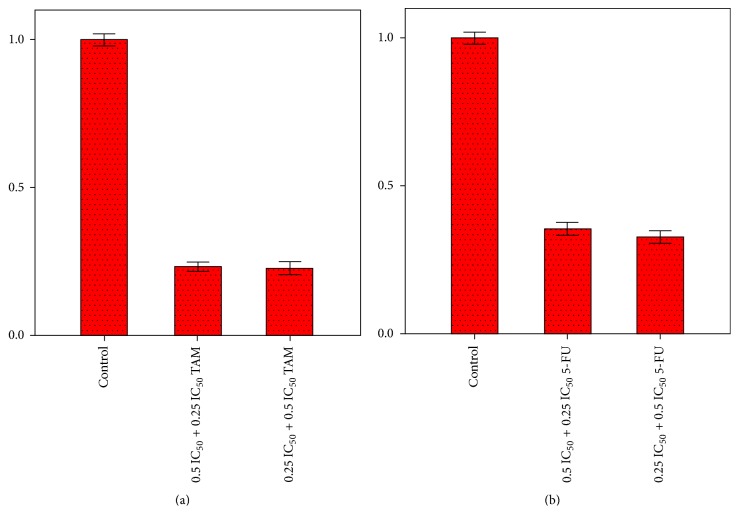
Effect of combination of compound** 9** with tamoxifen on MCF-7 cells proliferation (a) and with 5-fluorouracil on HCT116 cells proliferation (b).

Estrogen-dependent breast cancer represents 70% of all types of breast cancer. MCF-7 represents this type of cancer in which hormonal treatment (tamoxifen) is used. Combination therapy is used in order to prevent resistance or recurrence [21]. Study of the effect of combination of one of the most active analogues,** 9**, with tamoxifen on MCF-7 cells proliferation was carried out. Combination of half or quarter of IC_50_ value of compound** 9** with quarter or half of IC_50_ value of tamoxifen, respectively, inhibited the proliferation of MCF-7 cells by 77 and 76%, respectively, with combination index (CI) values of 0.25 ± 0.03 and 0.17 ± 0.06, respectively. Similar study was performed for evaluation of combination effect of compound** 9** with 5-fluorouracil on HCT116 cells proliferation (Figure 1). Combination of half or quarter of IC_50_ value of compound** 9** with quarter or half of IC_50_ value of 5-fluorouracil, respectively, inhibited the proliferation of HCT116 by 65 and 68%, respectively, with CI values of 0.37 ± 0.07 and 0.2 ± 0.06, respectively. All CI values were found to be <1, meaning that compound** 9** exerts synergistic effects with tamoxifen and 5-fluorouracil against MCF-7 and HCT116 cells proliferation, respectively.

#### 2.2.3. Cell Cycle Analysis

**Figure 2:**
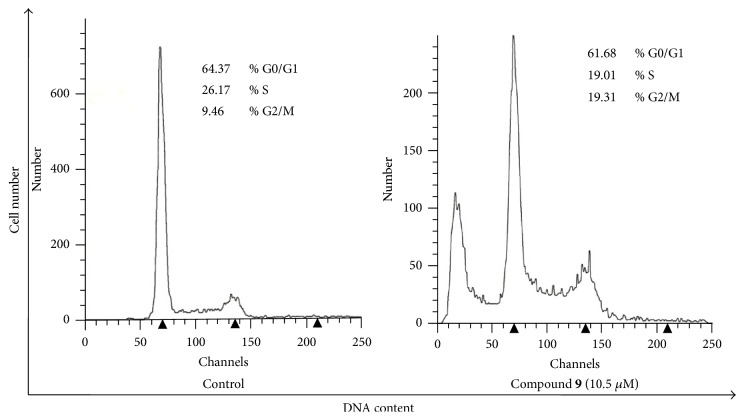
Cell cycle analysis showing the effect of compound** 9** on cell cycle progression in HCT116 cells.

Analogue** 9** was subjected to a cell cycle analysis to investigate whether its mechanism of action was similar to griseofulvin** 1** and the 2′-benzyloxy analogue** 3**. Both** 1** and** 3** have previously been confirmed to exhibit antiproliferative effect on human cancer cells and to cause G2/M arrest [13, 18]. Cell cycle analysis of HCT116 cells treated with compound** 9** (10.5 *μ*M) was performed by flow cytometry using propidium iodide (PI) staining. As evident from Figure 2, analogue** 9** induced G2/M arrest. Based on these results and as expected, we concluded that analogue** 9** works by similar mechanism of action to** 1** and** 3**.

#### 2.2.4. Effect of Compound** 9** on Apoptosis

**Figure 3:**
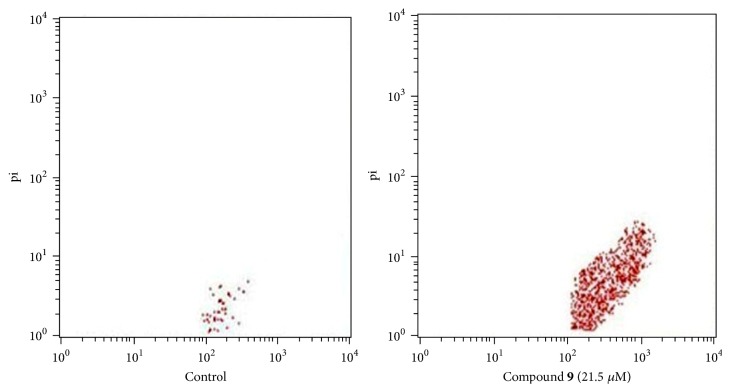
Flow cytometric evaluation of effect of compound** 9** on MCF-7 cells apoptosis.

In order to study the effect of compound** 9** on apoptosis, MCF-7 cells were treated with compound** 9** (21.5 *μ*M) for 48 hrs and harvested for fluorescence microscopic and flow cytometric analysis of Annexin V-FITC/propidium iodide (PI) staining. As shown in Figure 3, compound** 9** caused an appreciable increase in the percentage of apoptotic cells. The percentage of apoptotic cells was 2.65% in control untreated MCF-7 cells and 31.75% after treatment with compound** 9**.

### 2.3. Molecular Modeling

It was reported that griseofulvin** 1**, which binds to tubulin [10], shares its binding site in tubulin with taxol and kinetically suppresses microtubule dynamics in a similar manner [12]. Molecular docking simulation of the target compounds** 5**–**10** was performed into the active site of *α*/*β*-tubulin heterodimer (1JFF), which was obtained from Protein Data Bank, using Molecular Operating Environment (MOE®) version 2016.08 [22], to rationalize the obtained* in vitro* cytotoxicity results. Due to the geometrical isomeric nature of compounds** 5**–**10**, both *E* and *Z* isomers were docked independently.

**Figure 4:**
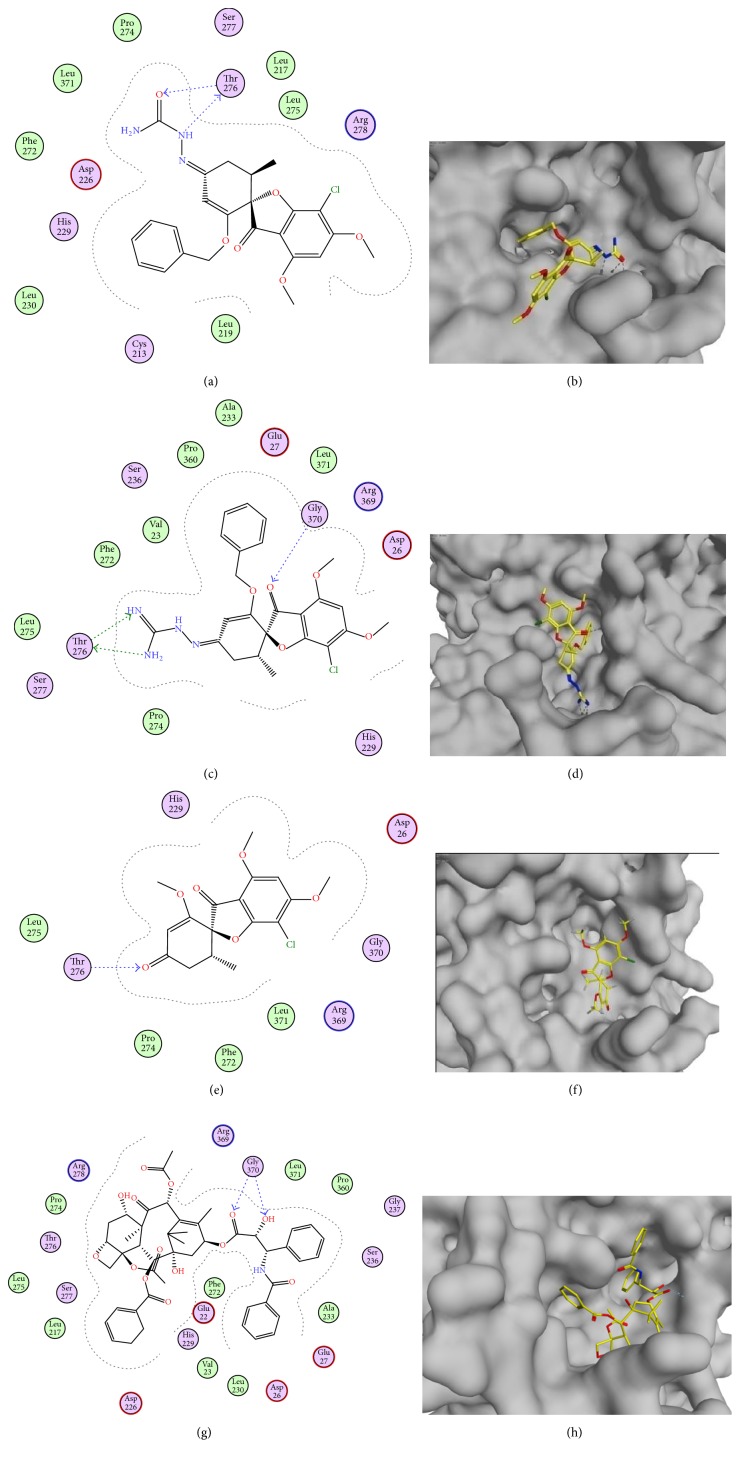
((a) and (b)) 2D and 3D representation of the binding mode of compound** 9** in the tubulin binding site. ((c) and (d)) 2D and 3D representation of the binding mode of compound** 10** in the tubulin binding site. ((e) and (f)) 2D and 3D representation of the binding mode of griseofulvin** (1)** in the tubulin binding site. ((g) and (h)) 2D and 3D representation of the binding mode of taxol in the tubulin binding site.

From the docking studies of the target compounds** 5**–**10** and their binding energy (Δ*G*) (Table 1), we can observe a rough correlation with the* in vitro* anticancer activity compared to that of** 1** and** 3**. The results also revealed that the substitution of 4′-carbonyl group of** 1** and** 3** with polar hydrophilic moieties increases binding affinity to tubulin through hydrogen bonding (Figure 4).

### 2.4. Calculated Physicochemical and ADMET Properties

The effect of the structural modification on the physicochemical and ADMET properties of** 1** and** 3** and consequently on their biological activity was studied. These properties were calculated using ACD/I-Lab [23].

Calculations displayed in Table 2 reveal the following. (i) First, all the synthesized analogues comply with Lipinski's rule of five and Veber rule. Hence, theoretically, all of these compounds should present good passive oral absorption. (ii) Second, because pKa determines the degree of ionization, it has a major effect on solubility in aqueous media. The added moieties impact a basic pKa value of 9.50 for compounds** 7** and** 10** and acidic pKa values of 3.6 for** 8** and 2.8 for** 5**. The added acidic and basic moieties have the ability of salt formation with a suitable counter ion and thus conferred increase in water solubility. (iii) Third, all the synthesized analogues revealed higher water solubility (log⁡*S*) than their parent ketones** 1** and** 3**. Compounds** 5**,** 7**,** 8**, and** 10** manifested the highest water solubility due to their ability of ionization. (iv) All tested compounds revealed comparable intestinal permeability to griseofulvin except compound** 7**, which had the least log⁡*P* value (1.93). And so all tested compounds had 100% human intestinal absorption except compound** 7** (78%). (v) All investigated compounds showed good oral bioavailability (30–70%), except compound** 5**, which revealed bioavailability less than 30%. (vi) The added hydrophilic moieties decreased the extent of brain penetration (log⁡BB) in all tested compounds. (vii) Compounds** 5** and** 9** had in silico toxicity risk profiles better than** 1**, while compounds** 6**,** 7**,** 8**, and** 10** had toxicity risk profiles similar to that of** 1**.

### 2.5. Solubility Measurement

Water solubility of the target compounds** 5**–**10** were tested in both pH 1.2 and 6.8 buffer solutions and compared with that of** 1** and** 3**. After determination of *λ*_max_ for each compound at pH 1.2 and 6.8 buffer solutions, equilibrium solubility of each compound at pH 1.2 and 6.8 buffer solutions was determined (Table 3).

All investigated compounds revealed higher solubility in pH 1.2 buffer solution than** 1** and** 3**; compounds** 7**,** 8**, and** 10** had the highest solubility values. The high solubility of** 7**,** 8**, and** 10** in pH 1.2 buffer solution was due to presence of the basic pyridine ring in** 8** and aminoguanidine moiety in** 7** and** 10**. In pH 6.8 buffer solution, all compounds manifested higher solubility than** 1** and** 3**. Compound** 5** showed the highest solubility, and this is elucidated by presence of the ionizable carboxyl group in its structure. The solubility results coincided to a large extent with the results of solubility (log⁡*S*) obtained from the previous calculated physicochemical properties.

## 3. Conclusion

Based on the good anticancer activity of griseofulvin analogues and its low water solubility, six new griseofulvin analogues were synthesized and screened for their antiproliferative activity. Analogues** 9** and** 10** were the most potent analogues against the cancer cell lines MCF-7 and HCT116 with IC_50_ values ranging from 8.39 to 21.50 *μ*M. Analogue** 9** was subjected to further study of effect of its combination with tamoxifen or 5-fluorouracil on proliferation of MCF-7 and HCT116 cells, respectively. Compound** 9** revealed synergistic activity with tamoxifen and 5-fluorouracil. In addition, compound** 9** induced apoptosis in MCF-7 cells and was confirmed to exert its anticancer effect through induction of G2/M cell cycle arrest* in vitro* as previously documented for both** 1** and** 3**. Further, a solubility study was performed and all synthesized analogues exhibited higher water solubility than their parent ketones** 1** and** 3** and this was in accordance with the data obtained through physicochemical calculations. Finally, substitution at position 4′ of griseofulvin** 1** or the more potent 2′-benzyloxy analogue** 3** with semicarbazide or aminoguanidine increased anticancer activity with improvement of water solubility.

## 4. Experimental Section

### 4.1. Chemistry

Reactions were monitored by TLC, silica gel 60 F_254_ precoated sheets, 20 × 20 cm, with layer thickness of 0.2 mm (E. Merck, Germany), and spots were visualized using UV-lamp at *λ*_max_ 254 nm. Column chromatography was performed using Fluka silica gel 60 (particle size: 0.063–0.02 mm). Melting points were determined on Stuart electrothermal melting point apparatus and were uncorrected. IR spectra were recorded as KBr disks on a Shimadzu IR 400-91527 spectrophotometer or on Thermo-912AO683 FT-IR. NMR spectra (60 MHz and 400 MHz for ^1^H and 100 MHz for ^13^C) were observed on Varian EM-360L NMR spectrophotometer (60 MHz) or Bruker Avance III HD FT‐high-resolution NMR, 400 MHz, with tetramethylsilane as the internal standard. Chemical shifts (*δ*) values are given in parts per million (ppm) using DMSO-d_6_ and CDCl_3_ as solvents. Elemental analysis was performed on apparatus from Analysensysteme GmbH, Hanau, Germany.

#### 4.1.1. Synthesis of (2S,6′R)-7-Chloro-4,6-dimethoxy-6′-methyl-3H-spiro[benzo-furan-2,1′-cyclohexane]-2′,3,4′-trione** (2)**

Griseofulvin** 1** (14.2 mmol) was dissolved in glacial acetic acid (25 ml) by heating and stirring on a water bath. 2 N aqueous sulfuric acid (5 ml) was added, and the clear solution was heated on the water bath and stirred for 1 hour. White precipitate of the product began to separate after a few minutes. The reaction mixture was allowed to cool to room temperature; then water (50 ml) was added to the reaction mixture. Solid was filtered under suction, washed with methanol (3 × 5 ml) and ether (5 ml) and dried, and then crystallized from methanol to afford the desired pure product. Yield: 4.36 g, 91%,* m.p.* 262-263°C as reported [20].

^1^H-NMR (60 MHz, DMSO-d_6_): 6.3 (s, 1H), 5.3 (s, 1H), 4.0 (s, 3H), 3.8 (s, 3H) 3.0–2.2 (m, 3H), 0.8 (d, *J* = 4, 3H).

#### 4.1.2. Synthesis of (2S,6′R)-2′-Benzyloxy-7-chloro-4,6-dimethoxy-6′-methyl-3H-spiro[benzofuran-2,1′-cyclohex[2′]en]-3,4′-dione** (3)**

A mixture of** 3** (11.8 mmol, 1 equiv) in dimethylformamide (40 ml) and anhydrous potassium carbonate (11.8 mmol, 2 equiv) was stirred for 30 minutes at room temperature. Benzyl bromide (17.62 mmol, 1.5 equiv) was added and stirring was continued for 16 hours at the same temperature. Sodium carbonate solution 10% (50 ml) was added to the reaction mixture; then the mixture was extracted with ethyl acetate (80 ml). The organic phase was washed with sodium carbonate solution [10%] (2 × 30 ml) and then with brine (30 ml). The organic phase was dried over anhydrous magnesium sulfate and then evaporated under vacuum.

The residue was purified by silica gel column chromatography using n-hexane : ethyl acetate (7 : 3) as eluent to afford the desired product. Yield: 1.0 g, 20%,* m.p.*: 162-163°C as reported [24]. ^1^H-NMR (60 MHz, CDCl_3_): *δ* 7.5 (s, 5H), 6.4 (s, 1H), 5.8 (s, 1H), 5.2 (s, 2H), 4.3 (s, 3H), 4.2 (s, 3H), 3.4–1.9 (m, 3H), 1.3 (d, *J* = 6 Hz, 3H).

#### 4.1.3. Synthesis of (E/Z)-(2S,6′R)-2′-Benzyloxy-7-chloro-4′-(hydroxylimino)-4,6-dimethoxy-6′-methyl-3H-spiro[benzofuran-2,1′-cyclohex [2′]en]-3-one** (4)**

Hydroxylamine hydrochloride (6.3 mmol, 3 equiv) and anhydrous sodium acetate (6.3 mmol, 3 equiv) were added to a solution of** 3** (2.1 mmol, 1 equiv) in super dry ethanol (30 ml). The mixture was refluxed for 3 hours, allowed to cool to room temperature, and diluted with methylene chloride (30 ml). The mixture was washed with distilled water (2 × 20 ml) and then brine (20 ml). The organic phase was dried over anhydrous magnesium sulfate. The organic layer was evaporated under vacuum. The residue was purified by silica gel column chromatography using n-hexane : ethyl acetate (6 : 4) as eluent to afford the desired product. Pale yellow, yield: 0.72 g, 78%,* m.p.* 139–141°C as reported [17].

^1^H-NMR (60 MHz, CDCl_3_): *δ* 8.1 (s, 1H), 7.3 (s, 5H), 6.4 (s, 0.5H), 6.2 (s, 1H), 5.7 (s, 0.5H), 4.9–4.7 (2H, m), 4.1 (s, 3H), 4.0 (s, 3H), 3.2–2.1 (m, 3H), 1.0 (d, *J* = 6 Hz, 3H).

#### 4.1.4. Synthesis of 2-(((E/Z)-[(2S,6′R)2′-Benzyloxy-7-chloro-4,6-dimethoxy-6′-methyl-3-oxo-3H-spiro[benzofuran-2,1′-cyclohex[2′]en]-4′-ylidene]amino)oxy) Acetic Acid** (5)**

A solution of** 4** (0.56 mmol, 1 equiv) and sodium hydride 60% dispersion in mineral oil (1.12 mmol, 2 equiv) in dimethylformamide (20 ml) was stirred at room temperature for 30 min. Chloroacetic acid (1.12 mmol, 2.0 equiv) was added to the reaction mixture and stirring continued for 12 hours at the same temperature. Water (30 ml) was added and the mixture was washed with methylene chloride (2 × 20 ml). The aqueous layer was acidified to pH 4 with hydrochloric acid and then extracted with ethyl acetate (3 × 15 ml). The combined organic phase was dried over anhydrous magnesium sulfate and then evaporated under vacuum. The residue was purified by silica gel column chromatography using ethyl acetate : methanol : glacial acetic acid (9 : 0.9 : 0.1) as eluent to afford the desired product. Pale yellow, yield: 0.18 g, 63%,* m.p.* 155–157°C.

IR (KBr, cm^−1^): 1604, 1695, 2555–3500. ^1^H-NMR (400 MHz, DMSO-d_6_): *δ* 7.30–7.14 (m, 5H), 6.46 (s, 1H), 6.33 (s, 0.5H), 5.69 (s, 0.5H), 5.00–4.84 (m, 2H), 4.11 (s, 2H), 4.03 (s, 3H), 3.93 (s, 3H), 3.04–3.01 (m, 0.5H), 2.76–2.69 (m, 0.5H), 2.50–2.43 (m, 1H), 2.35–2.31 (dd, *J* = 4, 4 Hz, 1H), 0.86 (d, *J* = 8 Hz, 3H). ^13^C-NMR (100 MHz, DMSO-d_6_): *δ* 193.2, 193.0, 171.9, 171.6, 169.0, 164.6, 158.8, 157.7, 156.1, 152.0, 148.3, 136.7, 136.4, 128.8, 128.7, 128.2, 128.1, 126.8, 105.1, 101.3, 95.6, 95.0, 91.3, 74.4, 74.2, 69.7, 69.5, 57.9, 56.9, 36.1, 35.0, 30.7, 26.5, 14.6, 14.3. Elemental analysis, calculated (found), for C_25_H_24_ClNO_8_ (%): C, 59.82 (59.96); H, 4.82 (4.89); N, 2.79 (2.87).

#### 4.1.5. General Procedure for Synthesis of Compounds** 6**,** 7**,** 9**, and** 10**

Semicarbazide hydrochloride [for** 6** and** 9**] or aminoguanidine hydrochloride [for** 7** and** 10**] (2.55 mmol, 3 equiv) and anhydrous sodium acetate (2.55 mmol, 3 equiv) were added to a solution of respective ketone** 1** or** 3** (0.85 mmol, 1 equiv) in super dry ethanol (30 ml). The mixture was refluxed for 8 hours, allowed to cool to room temperature, and diluted with water (50 ml). The mixture was extracted with methylene chloride (2 × 30 ml). The combined organic phase was dried over anhydrous magnesium sulfate and then evaporated under vacuum.

*(1) (E/Z)-2-((2S,6*′*R)-7-Chloro-2*′*,4,6-trimethoxy-6*′*-methyl-3-oxo-3H-spiro[benzofuran-2,1*′*-cyclohex[2*′*]en]-4*′*-ylidene)hydrazine-1-carboxamide ****(6)***. The residue was purified by silica gel column chromatography using n-hexane : ethyl acetate (3 : 7) as eluent. White, yield: 0.26 g, 74%,* m.p.* 213–215°C. FT-IR (KBr, cm^−1^): 1614, 1645, 1701, 2965, 3369, 3395, and 3512. ^1^H-NMR (400 MHz, DMSO-d_6_): *δ* 9.56 (s, 0.5H), 9.20 (s, 0.5H), 6.47 (s, 1H), 6.35 (s, 1H), 6.27 (s, 1H), 6.23 (s, 0.5H), 5.67 (s, 0.5H), 4.03 (s, 3H), 3.93 (s, 3H), 3.58 (s, 1.5H), 3.46 (s, 1.5H), 2.81–2.72 (m, 1H), 2.50–2.43 (m, 1H), 2.37–2.33 (m, 1H), 0.80 (d, *J* = 8 Hz, 3H). ^13^C-NMR (100 MHz, DMSO-d_6_): *δ* 193.2, 193.0, 168.9, 164.6, 160.3, 157.8, 157.7, 157.2, 145.0, 141.4, 105.0, 103.5, 95.6, 95.4, 91.4, 91.3, 91.1, 91.0, 57.9, 56.9, 56.4, 56.2, 36.3, 35.2, 27.8, 14.6, 14.4. Elemental analysis, calculated (found), for C_18_H_20_ClN_3_O_6_ (%): C, 52.75 (52.89); H, 4.92 (4.95); N, 10.25 (10.42).

*(2) 2-((2S,6*′*R)-7-Chloro-2*′*,4,6-trimethoxy-6*′*-methyl-3-oxo-3H-spiro[benzo-furan-2,1*′*-cyclohex[2*′*]en]-4*′*-ylidene)hydrazine-1-carboximidamide ****(7)***. The residue was purified by silica gel column chromatography using n-hexane : ethyl acetate (2 : 8) as eluent. White powder, yield: 0.29 g, 63%,* m.p.* 198–201°C. IR (KBr, cm^−1^): 1603, 1687, 2935, 3210, and 3430. ^1^H-NMR (400 MHz, DMSO-d_6_): *δ* 7.58 (s, 4H), 6.49 (s, 1H), 5.90 (s, 1H), 4.04 (s, 3H), 3.94 (s, 3H), 3.57 (s, 3H), 3.07 (m, 1H), 2.92 (dd, *J* = 4, 4 Hz, 1H), 2.61–2.58 (m, 1H), 0.83 (d, *J* = 8 Hz, 3H). ^13^C-NMR (100 MHz, DMSO-d_6_): *δ* 192.7, 168.9, 164.8, 159.9, 157.8, 156.2, 151.9, 104.8, 102.1, 95.6, 91.5, 90.2, 58.0, 57.0, 56.5, 35.1, 28.7, 14.5. Elemental analysis, calculated (found), for C_18_H_21_ClN_4_O_5_ (%): C, 52.88 (53.04); H, 5.18 (5.16); N, 13.70 (13.96).

*(3) 2-((2S,6*′*R)-2*′*-Benzyloxy-7-chloro-4,6-dimethoxy-6*′*-methyl-3-oxo-3H-spiro[benzofuran-2,1*′*-cyclohex[2*′*]en]-4*′*-ylidene)hydrazine-1-carboxamide ****(9)***. The residue was purified by silica gel column chromatography (n-hexane : ethyl acetate/3 : 7) to afford the desired product. Pale yellow powder, yield: 0.24 g, 71%,* m.p.* 171–173°C. FT-IR (KBr, cm^−1^) 1612, 1701, 2928, 3200, 3467. ^1^H-NMR (400 MHz, DMSO-d_6_): *δ* 9.22 (s, 1H), 7.31–7.16 (m, 5H), 6.46 (s, 1H), 6.26 (s, 2H), 5.75 (s, 1H), 4.95–4.83 (m, 2H), 4.03 (s, 3H), 3.92 (s, 3H), 2.78–2.69 (m, 1H), 2.51–2.41 (m, 1H), 2.37–2.34 (m, 1H), 0.89 (d, *J* = 4 Hz, 3H). ^13^C-NMR (100 MHz, DMSO-d_6_), *δ*193.2, 169.0, 164.5, 157.7, 157.5, 156.0, 145.0, 136.7, 128.9, 128.8, 128.3, 128.1, 126.9, 105.1, 104.7, 95.6, 91.3, 91.1, 69.5, 57.9, 56.9, 35.1, 27.9, 14.6. Elemental analysis, calculated (found), for C_24_H_24_ClN_3_O_6_ (%): C, 59.32 (59.51); H, 4.98 (5.07); N, 8.65 (8.82).

*(4) 2-((2S,6*′*R)-2*′*-Benzyloxy-7-chloro-4,6-dimethoxy-6*′*-methyl-3-oxo-3H-spiro[benzofuran-2,1*′*-cyclohex[2*′*]en]-4*′*-ylidene)hydrazine-1-carboximidamide ****(10)***. The residue was purified by silica gel column chromatography (n-hexane : ethyl acetate/2 : 8) to afford the desired product. Pale yellow powder, yield: 0.20 g, 59%,* m.p.* 162–164°C. IR (KBr, cm^−1^): 1603, 1693, 2930, 3160, and 3370. ^1^H-NMR (400 MHz, DMSO-d_6_), *δ* 7.40 (s, 4H), 7.29–7.18 (m, 5H), 6.47 (s, 1H), 6.36 (s, 1H), 5.09 (dd, *J* = 12, 12 Hz, 2H), 4.03 (s, 3H), 3.93 (s, 3H), 3.20 (m, 1H), 2.94 (m, 1H), 2.60 (m, 1H), 0.89 (d, *J* = 8 Hz, 3H). ^13^C-NMR (100 MHz, DMSO-d_6_): *δ* 192.4, 169.0, 164.7, 162.0, 157.9, 156.0, 149.7, 135.8, 128.9, 128.5, 127.1, 104.8, 102.1, 95.6, 91.6, 90.8, 70.4, 58.0, 57.0, 36.3, 33.8, 14.4. Elemental analysis, calculated (found), for C_24_H_25_ClN_4_O_5_ (%): C, 59.44 (59.72); H, 5.20 (5.28); N, 11.55 (11.74).

#### 4.1.6. Synthesis of (*E*/*Z*)-N′-((2S,6′R)-7-Chloro-2′,4,6-trimethoxy-6′-methyl-3-oxo-3*H*-spiro[benzofuran-2,1′-cyclohex[2′]en]-4′-ylidene)isonicotinic Acid Hydrazide** (8)**

Few drops of glacial acetic acid were added to a solution of** 1** (0.85 mmol, 1 equiv) and isoniazid (1.70 mmol, 2 equiv) in anhydrous methanol (30 ml) to adjust the pH at about 5. The mixture was refluxed for 5 hours and allowed to cool to room temperature. The solvent was evaporated under vacuum. The residual solid was recrystallized from methanol. Pale yellow crystals, yield: 0.33 g, 82%,* m.p.* 133–135°C.

FT-IR (KBr, cm^−1^): 1615, 1650, 1701, and 3213. ^1^H-NMR (400 MHz, DMSO-d_6_): *δ* 11.04 (s, 0.4H), 10.86 (s, 0.6H), 8.74 (s, 2H), 7.77 (s, 2H), 6.49 (s, 1H), 6.33 (s, 0.4H), 5.86 (s, 0.6H), 4.05 (s, 3H), 3.95 (s, 3H), 3.65 (s, 1.5H), 3.61 (s, 1.5H), 2.99–2.89 (m, 1H), 2.72–2.64 (m, 1H), 2.51–2.40 (m, 1H), 0.85 (d, *J* = 8 Hz, 3H). ^13^C-NMR (100 MHz, DMSO-d_6_): *δ* 192.8, 169.0, 164.7, 162.7, 162.4, 160.5, 157.8, 153.9, 150.5, 141.9, 141.5, 122.2, 105.0, 103.1, 95.7, 91.5, 90.9, 58.0, 56.9, 56.7, 36.3, 35.5, 34.3, 28.8, 14.5, 14.4. Elemental analysis, calculated (found), for C_23_H_22_ClN_3_O_6_ (%): C, 58.54 (58.79); H, 4.70 (4.76); N, 8.90 (9.12).

### 4.2. Biological Investigations

#### 4.2.1. Cytotoxicity Assay

Breast carcinoma MCF-7 and colorectal cancer HCT116 cell lines were used in this study. Cancer cell lines were obtained frozen in liquid nitrogen (−180°C) from the American Type Culture Collection (ATCC). The tumor cell line was maintained by serial subculturing in RPMI 1640 media containing 10% bovine serum albumin at the National Cancer Institute, Cairo, Egypt.

Cytotoxicity assay was carried out according to the reported literature [25], where the sensitivity of the MCF-7 and HCT116 cell lines to the tested compounds and their combination was determined by the SRB assay. In brief, cells were seeded at a density of 3 × 10^3^ cells/well in 96-well microtiter plates. Cells were left to attach for 24 hours before incubation with drugs. Next, they were treated with different concentrations of the tested compounds (10, 20, 30, 40, 50, and 100 *μ*M).

For each sample, three wells were used and incubation was continued for 48 hours. Control cells containing 200 *μ*l/well of DMSO (0.1% v/v) were used similarly. At the end of incubation, cells were fixed with 20% trichloroacetic acid (TCA), stained with 0.4% Sulforhodamine B (SRB), and rinsed with 1% acetic acid. The bound protein stain was solubilized with Tris base (10 mM, pH 10.5) and the optical density (OD) of each well was measured spectrophotometrically at 570 nm using ELISA microplate reader (TECAN, Sunrise^TM^, Germany). The fraction of cell survival was calculated as follows.

Survival fraction = OD treated/OD control. The IC_50_ values (the concentrations that produce 50% inhibition of cell growth) were calculated using sigmoidal dose response curve-fitting models (GraphPad Prism software, version 5). Each experiment was repeated 3 times.

#### 4.2.2. Determination of Combination Index (CI)

The interaction between compound** 9** and either tamoxifen or 5-fluorouracil was evaluated by the isobologram analysis which is a dose-oriented geometric method of assessing drugs interaction. Two different combination regimens of compound** 9** with either tamoxifen on MCF-7 or 5-fluorouracil on HCT116 have been designed. In each regimen, half or quarter of IC_50_ values of compound** 9** combined with quarter or half of IC_50_ values of either tamoxifen or 5-fluorouracil, respectively. CI was employed to determine whether the compounds interacted synergistically, additively, or antagonistically. The degree of interaction between the two drugs was calculated using the combination index (CI), according to the isobologram equation [26]: CI = *d*1/*D*1 + *d*2/*D*2, where *d*1 and *d*2 signify the respective concentrations of compound** 9** and tamoxifen or 5-fluorouracil used in combination to produce a fixed level of inhibition, while *D*1 and *D*2 represent their concentrations that are alone able to produce the same magnitude of effect. If “CI” is less than 1, the effect of combination is synergistic, whereas if CI = 1 or >1, the effect is additive or antagonistic, respectively.

#### 4.2.3. Cell Cycle Analysis

MCF-7 cells from the treated (21.5 *μ*M of** 9**) and control cells were collected after 48 hours. Cell cycle distribution of the cell population was analyzed using CycleTEST™ Plus DNA Reagent Kit (BD Biosciences, USA). Cells were fixed with 70% ice-cold ethanol and washed and the pellet was suspended in trypsin buffer and left for 10 min at room temperature. 1% RNase buffer was added after addition of trypsin inhibitor and incubated for 10 min, followed by the addition of 100 *μ*g/ml propidium iodide. Samples were incubated in the dark for 30 min at 4°C. Distribution of cell-cycle phases with different DNA contents was determined using a FACScan flow cytometer (Becton-Dickinson, San Jose, CA, USA). This study was carried out at Cancer Biology Department, National Cancer Institute, Cairo, Egypt.

#### 4.2.4. Evaluation of Apoptosis Using Annexin V-FITC/PI-Stained Cells

In brief, untreated and treated MCF-7 cells (21.5 *μ*M of** 9**) were harvested and resuspended in calcium buffer at a concentration of 1 × 10^6^ cells/ml. Annexin V-FITC (10 *μ*l) was added to 100 *μ*l of cells. The tubes were incubated for 20 min in the dark. The cells were then washed with calcium buffer and propidium iodide (10 *μ*l) was added to each tube and incubated for at least 10 min on ice. Samples were analyzed by FACScan flow cytometer (Becton-Dickinson, San Jose, CA, USA) using CellQuest software (Becton-Dickinson, San Jose, CA).

### 4.3. Docking Simulations

The X-ray crystallographic structure of alpha-beta tubulin stabilized with taxol (PDB Id: 1JFF) was obtained from the Protein Data Bank through the internet (http://www.rcsb.org). All the molecular modeling calculations and docking simulation studies were performed at Medicinal Chemistry Department, Assiut University, using Molecular Operating Environment (MOE), version 2016.08 (Chemical Computing Group (CCG), Inc., Montreal, Canada) [22], on Dell Precision™ T3600 Workstation [Intel Xeon E5-1660, 3.3 GHz, 16 GB 1600 MHz DDR3, ECC RDIMM 1TB (7200 RPM), 1 GB NVIDIA Quadro 2000, Windows 7 Professional (64 Bit)].

### 4.4. Physicochemical and ADMET Properties Calculations

Physicochemical properties and ADMET calculations were performed using ACD/I-Lab online program [23].

### 4.5. Solubility Measurement

UV measurements were performed on single beam spectrophotometer (Jenway, model 6305, UK). Equilibrium solubility was performed through using digital precise shaking water bath (DAIHAN Scientific Co., model WSB-45, Republic of Korea).

#### 4.5.1. Preparation of Stock Solution and Determination of *λ*_max_

A powdered sample (20 mg), of each of the tested compounds [**3**,** 5**–**10**, and griseofulvin** 1**], was accurately weighed and dissolved in 100 ml of methanol to prepare 200 *μ*g/ml stock solution. Compound solutions (20 *μ*g/ml) in the investigated media (buffer solutions of pH 1.2 and 6.8) were prepared from stock solution after appropriate dilution. The prepared solutions were scanned in the UV-Vis region (200–800 nm) to determine the wavelength of maximum absorption (*λ*_max_) in each medium.

#### 4.5.2. Construction of Standard Calibration Curves

Solutions containing different concentrations of the investigated compound were prepared from stock solution after appropriate dilution with the investigated buffer solutions. The UV absorbance of the prepared sample solutions was measured at *λ*_max_ of the investigated compound using the investigated buffer solution as a reference solution (blank). The determined absorbance values were plotted versus the corresponding concentrations to construct the standard calibration curves.

#### 4.5.3. Determination of Equilibrium Solubility

Equilibrium solubility of each of the tested compounds was determined by placing an excess amount of the compound in stoppered glass volumetric flask containing 10 ml of the investigated buffer solution. The solutions were shaken at a rate of (40 ± 2) stroke/minute in a thermostatically controlled water bath at 37 ± 0.5°C for 24 hours to ensure equilibrium. Samples of 2 ml were withdrawn from each test solution, filtered immediately, and assayed spectrophotometrically at the determined *λ*_max_ of the investigated compound.

## Figures and Tables

**Table 1:** Interaction energies and *in vitro *cytotoxic activities of taxol, 5-fluorouracil, tamoxifen, griseofulvin **1**, compound **3**, and target compounds **5**–**10**.

Compound name/number	Isomer	Δ*G* (Kcal/mole)	IC_50_ (*μ*M) against HCT116	IC_50_ (*μ*M) against MCF-7
5-Fluorouracil	—	n.t.	19.50	n.t.
Tamoxifen	—	n.t.	n.t.	10.00
Taxol	—	−7.9426	n.t.	n.t.
Griseofulvin	—	−6.1399	35.50	69.00
**3**	—	−6.9434	35.80	32.80
**5**	*E*	−7.0208	>100	20.70
	*Z*	−7.1249		
**6**	*E*	−6.6714	87.84	55.87
	*Z*	−6.6542		
**7**	*E*	−6.7421	85.60	54.54
	*Z*	−6.7534		
**8**	*E*	−7.2516	39.80	25.30
	*Z*	−7.2894		
**9**	*E*	−7.3765	10.50	21.50
	*Z*	−7.3482		
**10**	*E*	−7.4810	8.39	14.50
	*Z*	−7.5143		

n.t.: not tested.

**Table 2:** Calculated physicochemical and ADME-Tox properties of the synthesized compounds **5**–**10** in addition to griseofulvin **(1)** and compound **3**.

ADME-Tox	**1**	**3**	**5**	**6**	**7**	**8**	**9**	**10**
Solubility (log⁡*S*)	−3.96	−5.52	−1.31 [pH = 6.8]	−3.25	−3.03 [pH = 6.8]	−3.85 [pH = 6.8]	−4.39	−4.13 [pH = 6.8]
			−4.81 [pH = 1.2]		−1.48 [pH = 1.2]	−1.99 [pH = 1.2]		−2.59 [pH = 1.2]
*F* (%)^a^	30–70% (0.637)	30–70% (0.637)	<30% (0.589)	30–70% (0.541)	30–70% (0.541)	30–70% (0.541)	30–70% (0.541)	30–70% (0.541)
HIA (%)^b^	100%	100%	100%	100%	78%	100%	100%	100%
Pe (cm/s)^c^	7.91 × 10^−4^	7.36 × 10^−4^	5.95 × 10^−4^	6.59 × 10^−4^	0.46 × 10^−4^	7.15 × 10^−4^	7 × 10^−4^	3.33 × 10^−4^
log⁡BB^d^ (log⁡*PS*)^e^	0.1	0.03	−0.55	−0.32	0.02	0.01	−0.28	−0.06
	(−1.4)	(−1.2)	(−2.6)	(−2.4)	(−3.5)	(−1.5)	(−1.7)	(−2.8)
pKa	—	—	2.80	—	9.50	3.60	—	9.50
LD_50_ mouse (mg kg^−1^, oral)	1000	1100	1300	810	560	850	1100	700
LD_50_ mouse (mg kg^−1^, intraperitoneal)	180	190	470	440	200	440	460	240
LD_50_ mouse (mg kg^−1^, intravenous)	100	62	96	10	25	130	54	21
LD_50_ mouse (mg kg^−1^, subcutaneous)	330	140	820	470	67	110	340	62
log⁡*P*^f^	2.51	3.79	3.96	2.02	1.93	3.35	3.67	3.55
TPSA (Å2)^g^	8.06	8.06	112.88	121.47	128.25	108.34	121.47	128.25
MW^h^	352.77	428.86	501.91	409.82	408.84	48.89	485.92	484.94
NOHD^i^	0	0	1	3	4	1	3	4
NOHA^j^	6	6	9	9	9	9	9	9
NORB^k^	3	5	8	4	5	5	6	7

^a^Human oral bioavailability (probability). ^b^Human intestinal absorption. ^c^Permeability (human jejunum). ^d^Extent of blood brain barrier penetration. ^e^Rate of brain penetration. ^f^Calculated lipophilicity. ^g^Topological polar surface area. ^h^Molecular weight. ^i^Number of hydrogen bond donors. ^j^Number of hydrogen bond acceptors. ^k^Number of rotatable bonds.

**Table 3:** *λ*_max_ (nm) and equilibrium solubility of tested compounds **5**–**10** at pH 1.2 and 6.8 buffer solutions.

Compound	*λ*_max_ (nm)		Mean solubility (*μ*g/ml) ± SD	
	pH 1.2	pH 6.8	pH 1.2	pH 6.8
**1**	292	292	12.32 ± 0.29	12.37 ± 0.26
**3**	294.5	295.6	11.13 ± 0.37	11.09 ± 0.82
**5**	294	294	14.26 ± 0.42	27.94 ± 0.27
**6**	294.5	291.5	14.86 ± 0.12	14.51 ± 0.39
**7**	293	294	31.49 ± 0.72	18.32 ± 0.46
**8**	294.5	295.8	29.13 ± 0.72	12.53 ± 0.54
**9**	292.5	293.5	13.97 ± 0.31	13.78 ± 0.07
**10**	290.5	294.6	26.67 ± 0.57	16.58 ± 0.58

## Supplementary Materials

Supplementary Materials
^13^C-chart of compound** 5**. ^13^C-chart of compound** 6**. ^13^C-chart of compound** 7**. ^13^C-chart of compound** 8**. ^13^C-chart of compound** 9**. ^13^C-chart of compound** 10**.
