# Supplementary material for: Design, Synthesis, and Cytotoxicity Evaluation of Novel Griseofulvin Analogues with Improved Water Solubility
Source: Int J Med Chem. 2017 Dec 7;2017:7386125. doi: 10.1155/2017/7386125 (PMC5738580; doi:10.1155/2017/7386125)

^{13}C-chart of compound 5

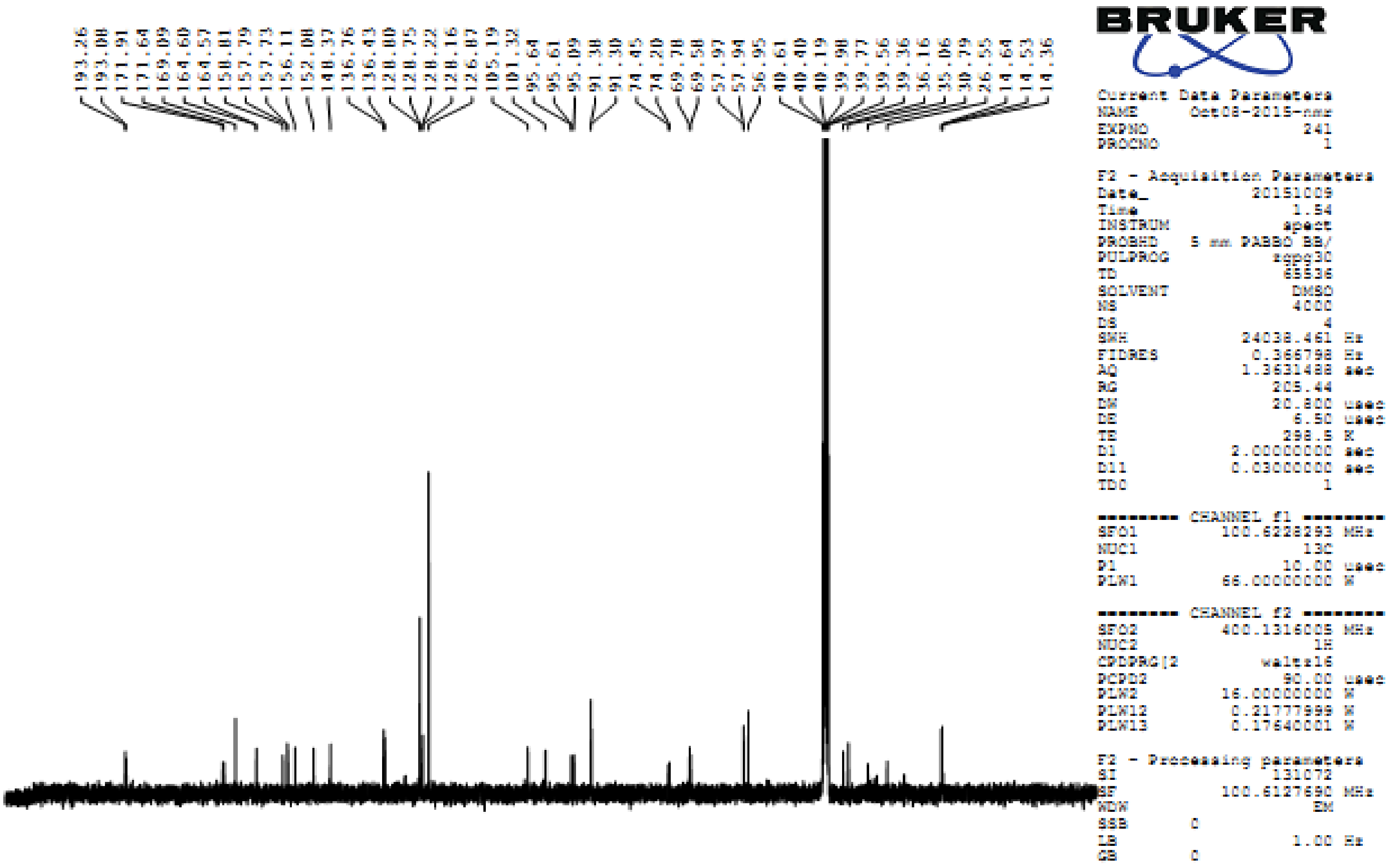

^{13}C-chart of compound 6

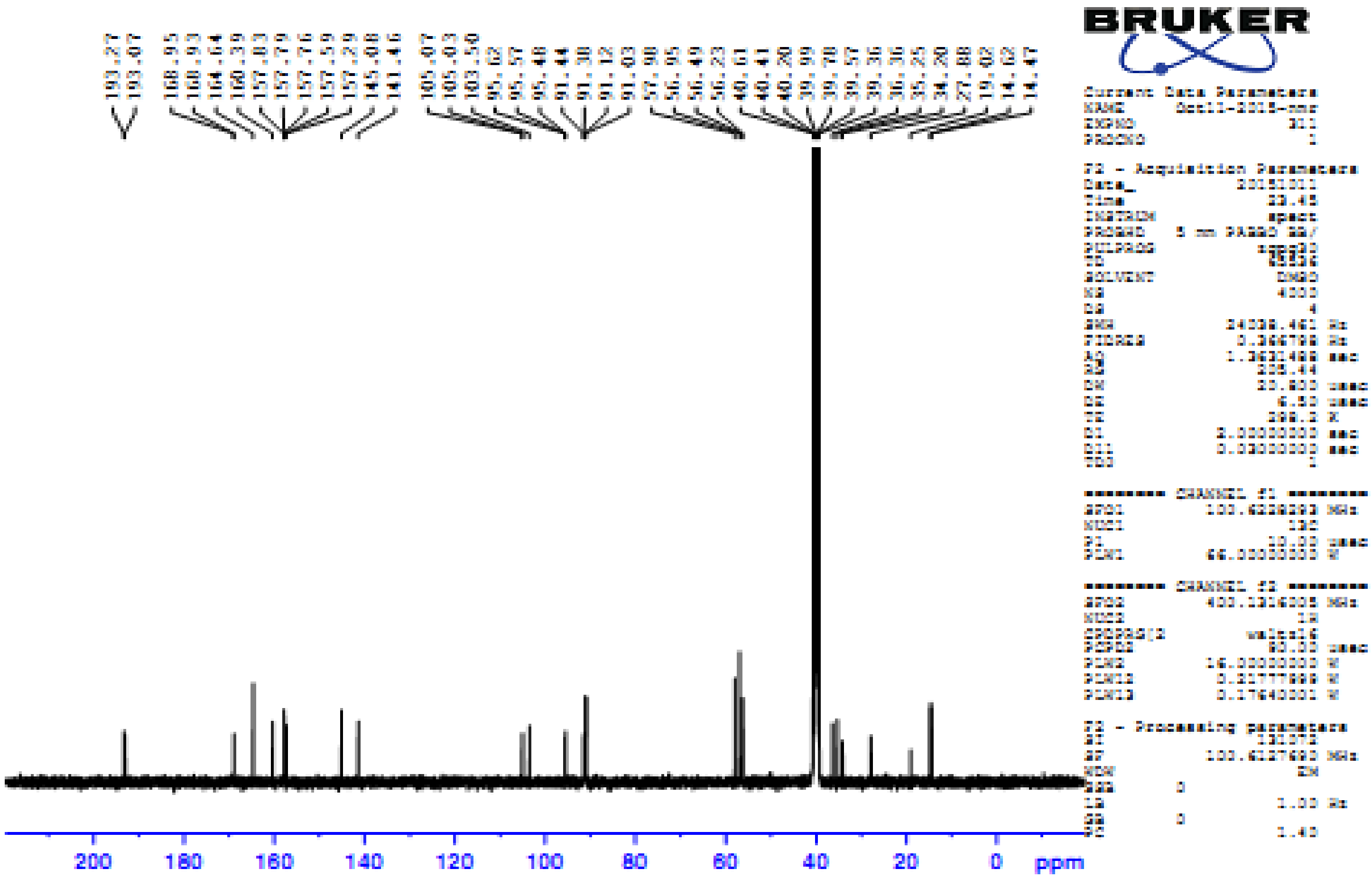

^{13}C-chart of compound 7

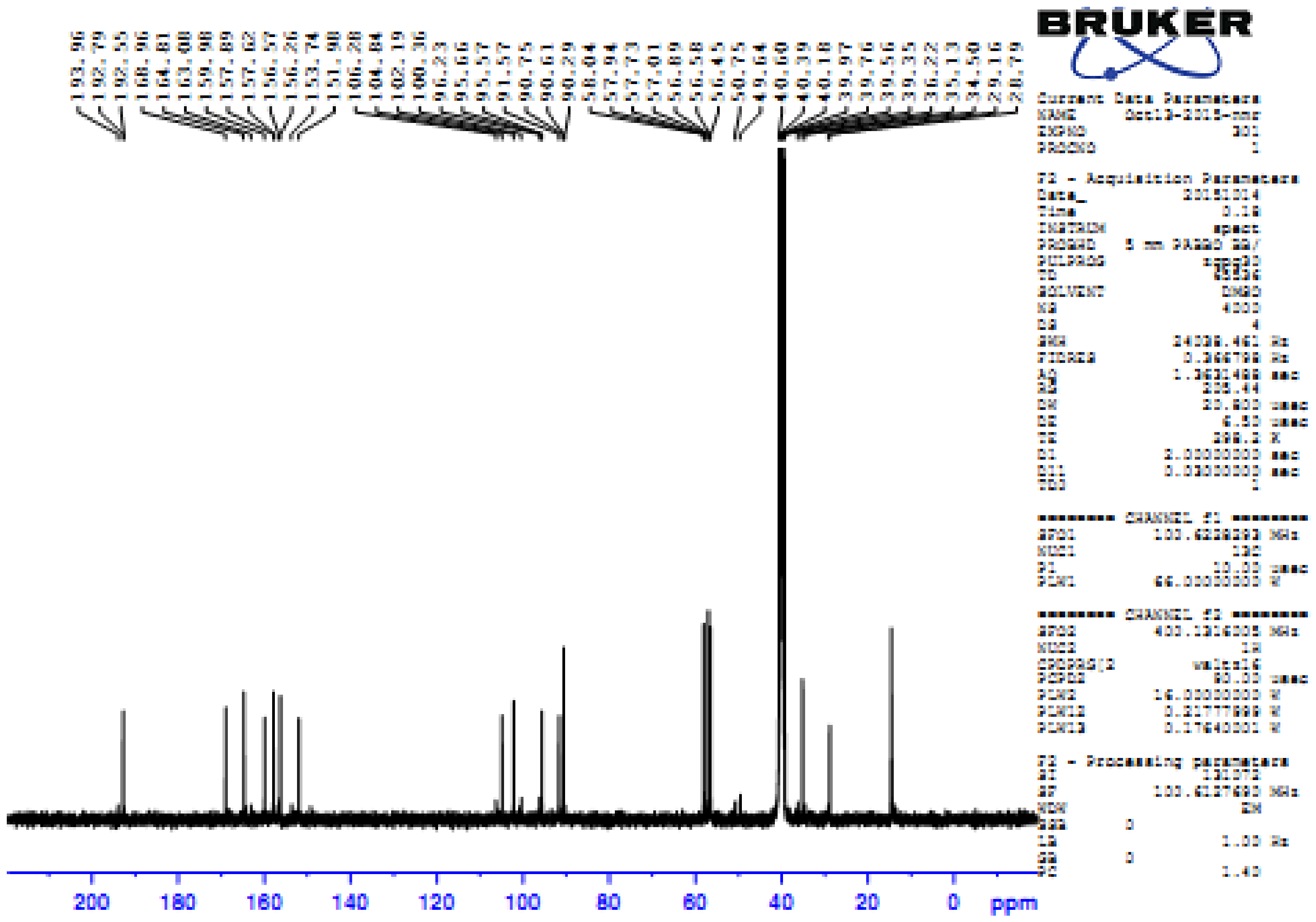

[13]C-chart of compound 8

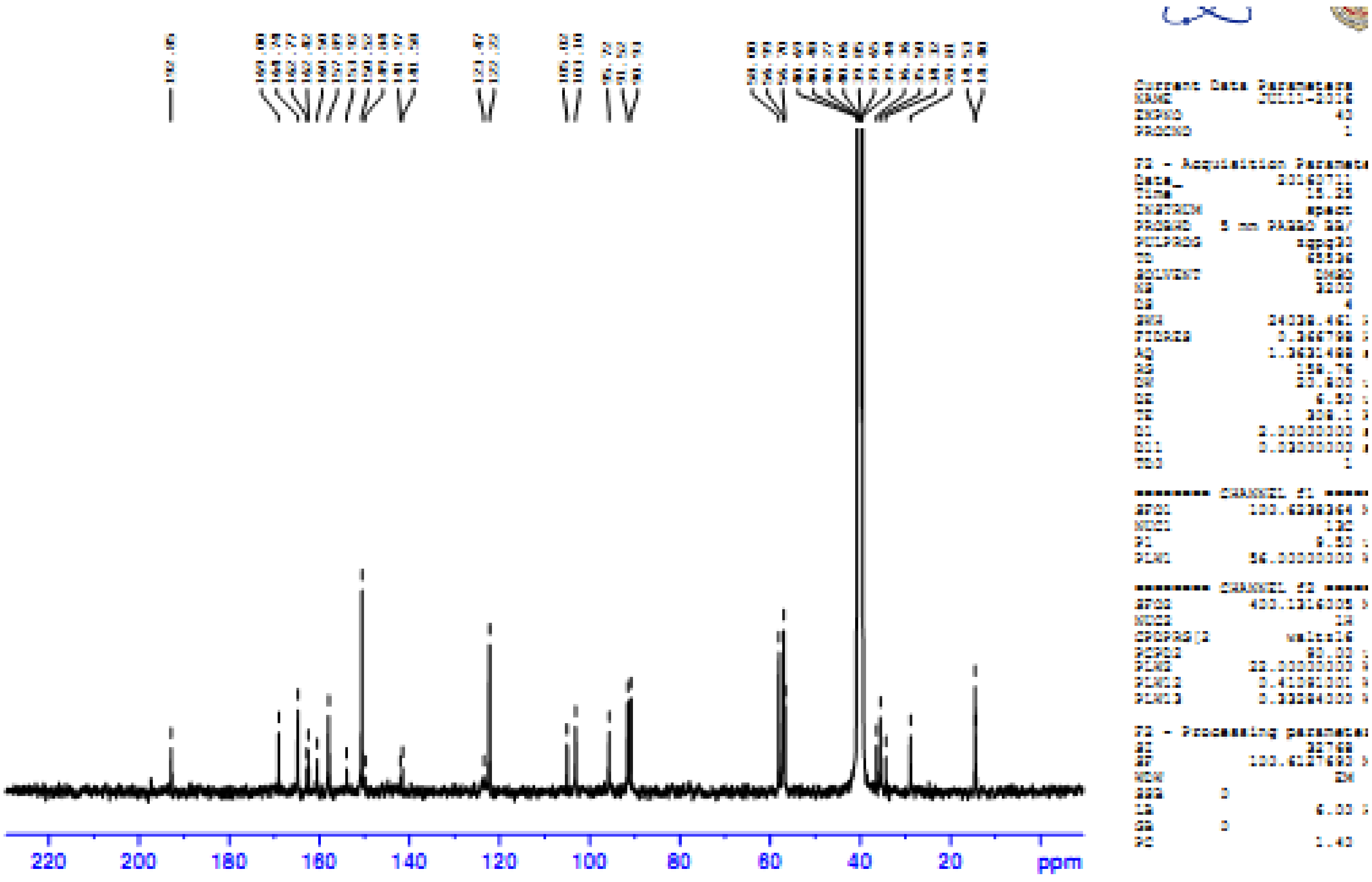

^{13}C-chart of compound 9

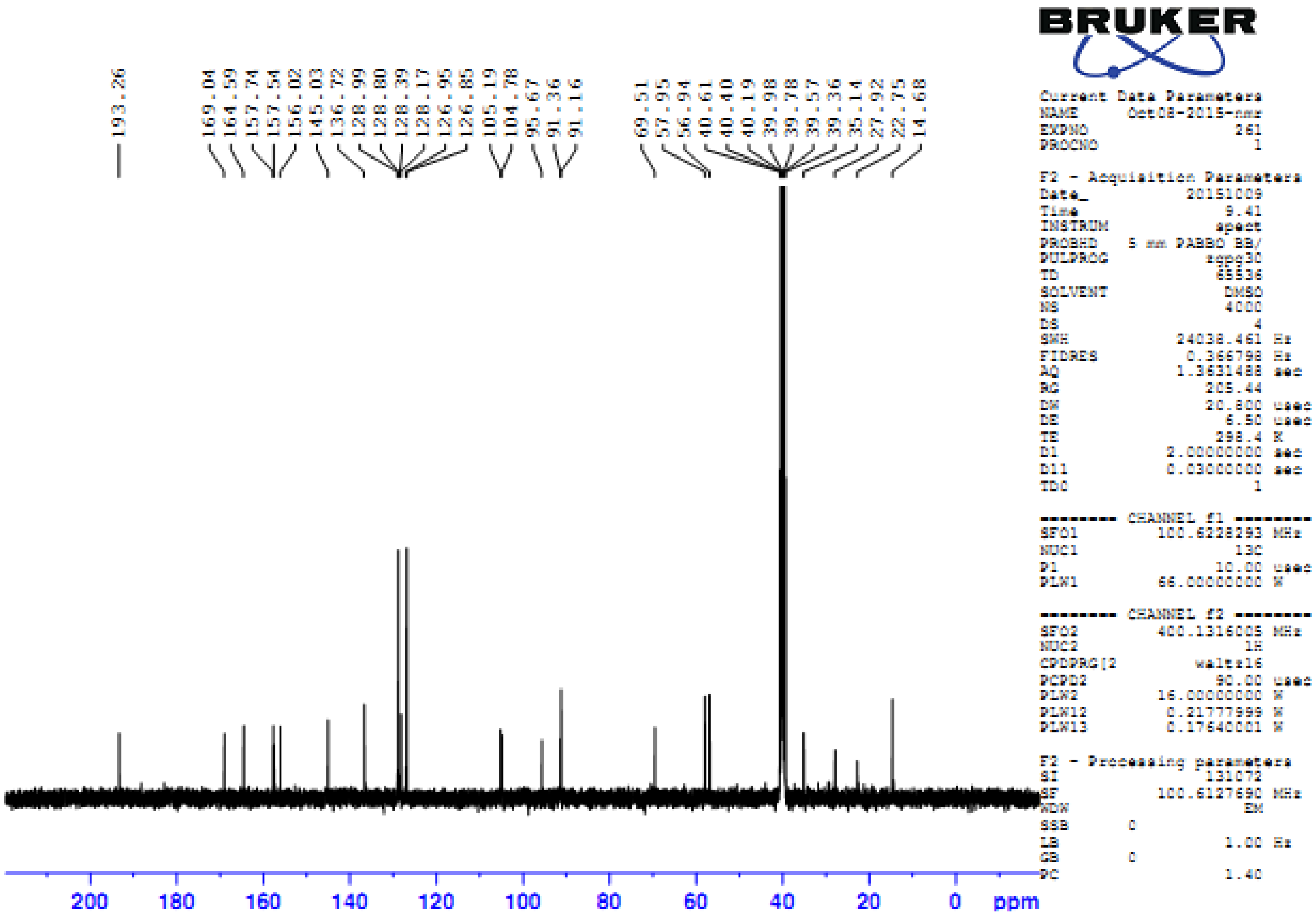

^{13}C-chart of compound 10

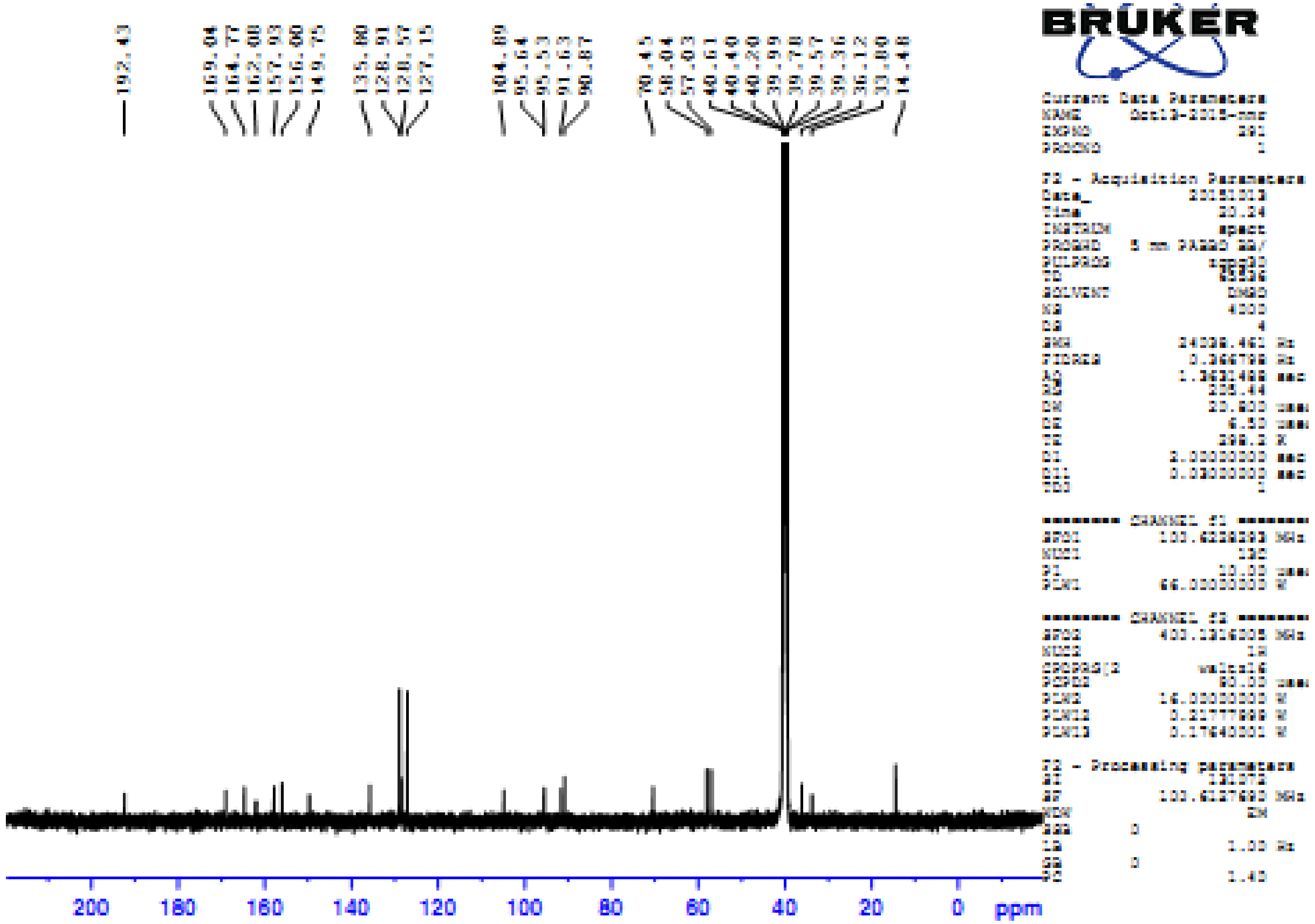

Supplement: Supplementary Materials — 13C-chart of compound 5. 13C-chart of compound 6. 13C-chart of compound 7. 13C-chart of compound 8. 13C-chart of compound 9. 13C-chart of compound 10. [file 7386125.f1.pdf]
